# Perceptions of social media harms and potential management strategies: vaping case study

**DOI:** 10.1186/s12889-024-18362-8

**Published:** 2024-03-21

**Authors:** Jonine Jancey, Gemma Crawford, Elizabeth Bowman, Katharina Wolf, Tama Leaver, Stella Bialous, Kahlia McCausland

**Affiliations:** 1 Collaboration for Evidence, Research and Impact in Public Health, School of Population Health, Curtin University, Kent Street, Perth, WA 6102 Australia; 2 School of Management and Marketing, Curtin University, Kent Street, Perth, WA 6102 Australia; 3 School of Media, Creative Arts and Social Inquiry, Curtin University, Kent Street, Perth, WA 6102 Australia; 4 School of Nursing, University of California, San Francisco, San Francisco, CA 94158 USA

**Keywords:** e-cigarettes, Vaping, Social media, Public health

## Abstract

**Background:**

The social media landscape is now ubiquitous in people’s everyday lives. It is a space where culture, politics, economics and sociological and public health discourses occur. There is mounting evidence that e-cigarette products are being promoted and advertised on social media, a media platform particularly popular with young people. Our research aimed to understand industry professionals’ perceptions of social media harms and potential management strategies using vaping as a case study.

**Methods:**

A critical realist perspective guided reflexive thematic analysis of the qualitative in depth, semi structured interviews. Data collection occurred in January and February 2023 with 13 participants working in the areas of public health, digital media, law, governance, tobacco control and advocacy.

**Results:**

Two superordinate themes emerged from the data: (1) Fathoming a complex system (social media) that contained the subordinate themes of Traversing Boundaries (crossing borders, crossing sectors) and Ungovernable (global and local landscapes, vested interests, self-regulation and opacity). (2) Addressing complexity (social media)– that contained the subordinate themes of Strengthening Institutions (global to local, policy and legislation, individuals and organisations); Defanging Industry (responsibility and transparency, moderation and algorithms, complaints); and Engaging Citizens (raising awareness, framing messaging).

**Conclusions:**

There was consensus among participants that e-cigarette related social media content can be harmful and government action is urgently needed. There was an identified need for the development of government led national-level regulatory frameworks, with government led appropriate legislation; identification of an organisation or organisations with suitable levels of regulatory power and resources to monitor, enforce and penalise noncompliant social media companies; accompanied by increased community awareness raising of harmful social media content and improved digital literacy.

## Introduction

The social media landscape is now ubiquitous in people’s everyday lives. Culture, politics, economics and sociological and public health discourses occur in this space [1]. In 2021, more than four billion people worldwide used social media, spending an average of 144 min each day on platforms such as TikTok, Twitter, Instagram, Facebook and YouTube [2]. These platforms provide users with opportunities to interact with a broad range of global content, exposing them to social change and marketing decisions, including harmful products such as e-cigarettes, an issue of global concern [3]. Globally, 82 million people were estimated to use e-cigarettes in 2021 [4], with the global e-cigarette market in 2023 estimated to be worth USD 24.6 billion and predicted to increase by 3.4% over the next five years [5].

E-cigarette products are known to be harmful to health [6]. Australia, where this research was centered, has historically taken a precautionary public health approach to e-cigarettes. Regulations have made it illegal to source liquid nicotine without a prescription from a medical doctor [7]. Yet, recent figures show that more than one-quarter (26.1%) of Australians aged 18–24 have tried e-cigarettes, with ‘ever-use’ [daily, weekly, monthly and less than monthly use], especially high among those who currently smoke (63.9%) [8]. Almost three-quarters (71.9%) of young people reported using e-cigarettes “out of curiosity,” and one in five (21.7%) used them because they believe that “vaping is less harmful than regular cigarette smoking” [8]. In response to increased e-cigarette uptake, in January 2024, Australian legislation was introduced banning the importation of single use vapes, with refillable vapes banned from March 2024 [9].

While traditional forms of e-cigarette advertising and promotion (print, radio and television) are regulated in Australia, tobacco and other independent vaping companies have increasingly turned their attention to social media platforms [10]. E-cigarette products are promoted and advertised on social media [11] through user-generated content, advertisements, and social media influencers [12–15]. The use of social media is particularly popular among young people [11]. There is substantial concern about young people’s exposure to e-cigarette advertising and user-generated content on social media, which is associated with lower perceptions of e-cigarette harm and more positive attitudes towards e-cigarettes, leading to the normalization of e-cigarettes and increased use [16, 17].

The World Health Organization’s (WHO) Framework Convention on Tobacco Control (FCTC) [18] was developed in response to the globalisation of the tobacco epidemic. The FCTC is a legally binding treaty with 183 signatories worldwide, that aims to reduce tobacco use and exposure to tobacco smoke. According to Article 13 of the FCTC, “*a comprehensive ban on all tobacco advertising, promotion and sponsorship applies to all forms of commercial communication, recommendation or action and all forms of contribution to any event, activity or individual with the aim, effect, or likely effect of promoting a tobacco product or tobacco use either directly or indirectly*.” This ban includes traditional media (print, radio and television) and social media [18]. However, social media companies are not bound by the FCTC, and the United States (US), which is home to many social media companies, is a non-party to the FCTC [19].

Currently, social media platforms self-regulate, guided by their own content policies, which refer to prohibited content, including advertising and promotion of tobacco and e-cigarette products. However, it appears these platforms are disregarding their own e-cigarette content policies by permitting non-compliant content to be posted, thereby exposing users to content they should not be exposed to [20, 21]. For example, there is evidence that social media account holders are not prevented from using various means to positively promote products on platforms (e.g., competitions encouraging users to share images of vape products, featuring vaping experiences, cross-promotion on alternate social networking platforms, and links to blogs to increase positive, searchable e-cigarette content) [22].

The self-regulation approach of social media platforms requires review as it is insufficient to control the content promoting e-cigarette products [14, 20]. Greater protection of social media users could be achieved by moving from self-regulation to public regulation (enforcement by an independent public regulator), which is legally binding to ensure greater accountability of social media platforms, content moderation, transparency, compliance and enforcement of content policy and sanctions for noncompliance [1]. Our research aimed to understand industry professionals’ perceptions of social media harms and potential management strategies using vaping as a case study. Although the research focused on Instagram and TikTok, findings could be extended to other social media platforms and other harmful products.

## Methods

### Methodology

We used a critical realist perspective to guide reflexive thematic analysis of the qualitative data [23]. This approach recognises that an individual’s experiences are socially located, having their own interpreted reality. Critical realism aims to provide a coherent interpretation of data anchored in the accounts of participants. This study was approved by the Curtin University Human Research Ethics Committee (HR2021-0250). The COREQ checklist [24] guided the reporting of findings.

### Research team

The study was conducted at a large public university located in Perth, Western Australia, with input from a collaborator in the US. The research team was composed of members with expertise in public health, social media, law, marketing, tobacco control, and qualitative research.

### Sampling

Eligible participants were from English-speaking countries; aged 18 years and older; were subject matter experts currently working in public health, digital media, law and governance, tobacco control and advocacy, providing a range of perspectives to enable exploration of the topic through multiple lenses.

### Recruitment

Seven members of the research team identified potential participants. Once identified, individuals were contacted via email, with any non-respondents being sent a follow-up email two weeks later. The email/s explained the purpose of the study by providing the participant with an information statement and consent form. Interested individuals were invited to contact the research team via email or telephone to express their interest in participating and have any questions answered. Prior to the interview, deidentified examples of e-cigarette-related posts from TikTok (videos) and Instagram (static images) were sent to the participants along with a summary of each platform’s content policies to orientate the participants to the topic and the discussion questions. Online interviews (Microsoft Teams) were arranged for a mutually convenient time.

### Data collection

The use of a semi-structured interview guide facilitated flexibility and adaptability within each interview [25]. The interview guide addressed the following: e-cigarette content on social media; management of e-cigarette content on social media; the development and enforcement of policy and regulations for e-cigarette content on social media; and demographic information (age, sex, type of work and workplace). An iterative process was applied. As new ideas and concepts were discovered, the concepts were integrated into subsequent data collection guiding further adaptation of the interview guide [26]. The interviews were conducted in English, lasted an average of 36 min (range 35–58 min) and were audio-recorded, with participant informed consent being obtained prior to commencement.

### Data analysis

All interviews were undertaken by a trained female researcher (EB), transcribed verbatim by the authors (JJ, EB) and checked for accuracy. Interview transcripts were anonymised and imported into NVivo (v12) to facilitate analysis. The primary author (JJ) initially coded the data. Initial codes were reviewed and discussed with a co-author (GC). Broad themes were constructed, then discussed, refined and confirmed by both authors (JJ and GC). This process facilitated immersion in both the data collection and analysis, thereby ensuring that the data coding adequately described the intentions and content of the interviews [27]. Reflexive thematic analysis included re-reading interviews to ensure familiarisation with the data, systematic coding of data, grouping of codes, identification of named themes, and refining themes [23]. As qualitative data looks for themes and patterns, it is important to ensure that the context and narrative are not lost by trying to quantify something that is not meant to be quantified. Accordingly, we did not quantify the data [28]. To increase the study rigour and trustworthiness of data, participants were emailed their transcript and draft results. This process provided them with an opportunity to provide feedback and check the accuracy of the presentation of findings (member checking). Demographic data were analysed using descriptive statistics (SPSS v26).

## Results

### Participants’ demographic profile

Thirteen participants agreed to participate in the one-on-one interviews. The participants were mainly female (*n* = 7), employed within a university (*n* = 9) or not-for-profit (NFP) organisations (*n* = 4), and resided in Australia (*n* = 9) or overseas (*n* = 4) (United Kingdom (UK) and US). Seven participants declined our invitation to participate due to time constraints and three did not respond to our email invitations.

### Findings

Two superordinate themes were constructed from the data: (1) Fathoming a complex system– social media; and (2) Addressing complexity– social media. To support the thematic analysis, de-identified quotes from participants were used in the presentation of the data.

#### Fathoming a complex system

**Fig. 1:**
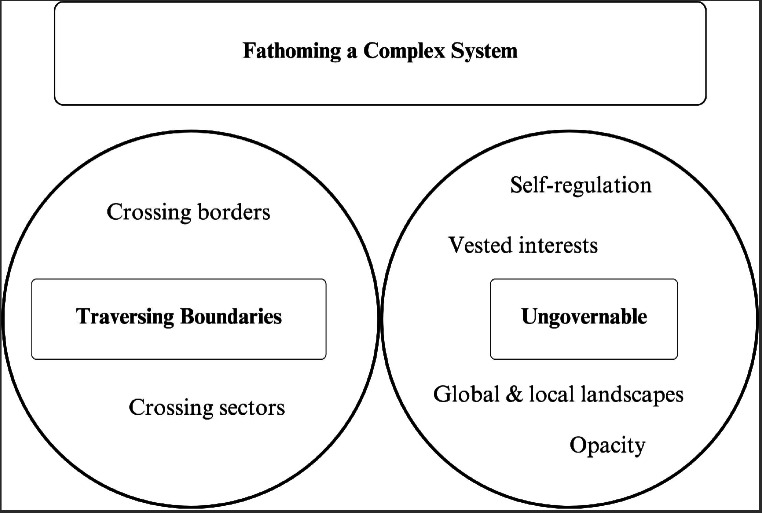
Fathoming a complex system (theme and subordinate themes)

Participants described the issues and challenges of navigating the social media landscape as it related to e-cigarettes. The subordinate themes of Traversing Boundaries (crossing borders, crossing sectors) and Ungovernable (global and local landscapes, vested interests, self-regulation and opacity) are presented in Fig. 1.

#### Traversing boundaries

##### Crossing borders

A major challenge of managing e-cigarette content on social media identified by participants was the porous borders that exist within and between countries and regions. These porous borders facilitate global information dissemination and sharing. This reach and cross border transmission of content has been enabled by the extensive global networks and abundant resources of transnational social media companies.

*… so, this global reach… just increases the complexities and makes it so much harder* [to manage social media content].” (Participant 1: Public health; Australian (Aus) university).

Participants suggested that the ability of posted content to traverse borders was further facilitated by social media users who willingly shared content, because it is well-marketed, targeted, appealing, and seen as an interactive opportunity. This was highlighted by one participant who noted the following:

*It’s really hard and tough to try and control social media because it transcends international borders and because a lot of what these companies are doing is trying to get people to pass things on, which they may be doing innocently because they’re, you know, fun, brightly coloured, attractive images. But the marketing is being done, and you know, by people* (account holders) *that don’t really understand what they’re getting into and what they’re part of, and sometimes for free.”* (Participant 10: Tobacco control; UK NFP).

##### Crossing sectors

Participants reported that the management of social media content was more challenging, due to the number of sectors involved in and affected by social media company activities. Nominated sectors included health, business, media, communication, and law. In Australia, this was exemplified as a matter for cross-portfolio consideration, as one participant noted.

*It’s a health issue, but actually, it’s a media issue. And the Communications Minister should be the one to look at it because she’s also got an alternative approach, which is to say social media companies are content service providers under the Telecommunications Act. She can make a content service provider rule, which would be managed by the Australian Communications and Media Authority [ACMA], or enforced by the ACMA, and that rule could be a prohibition against vaping advertising.”* (Participant 8: Governance and telecommunications; Aus university).

Social media regulation and policy were highlighted as convoluted and unclear. This led to questions regarding who is, and who should be responsible for the management of social media companies and in turn their content, “*there’s this whole discussion coming out of the ACCC* [Australian Competition and Consumer Commission] *… and I think one of the questions they’re putting out there is… which entity should be responsible?*” (Participant 12: Consumer advocacy; Aus NFP).

#### Ungovernable

##### Global and local landscapes

Participant narratives suggested that cross-border exposure to e-cigarette content on social media is exacerbated by the differing regulatory and legal frameworks globally, within and between countries and regions. For example, participants suggested that more profuse e-cigarette social media content is generated in countries with liberal tobacco control regulations; however, this same content is also viewed in countries with tighter regulations, such as Australia.

… *even if you had great laws… what about the fact that you can access these ads from other countries.”* (Participant 4: Public Health; Aus university).

Participants suggested that as a consequence of social media’s relative newness and pervasiveness, the development and implementation of regulations and legislation to manage social media companies was lacking and “*way out of date.”* For example:

*There is a pretty pressing need to modernise our legislation on all forms of tobacco and related marketing. So, at the moment we’re operating legislation that’s 30-odd years old.”* (Participant 4: Public health; Aus university).

##### Vested interests

Participants noted a range of actors with vested interests that included the tobacco and vaping industry, proponents of vaping, influencers, and the social media platforms themselves. The actors’ actions were at times recognised as conflicting with social media platform content controls, and that these actions directly benefited them (i.e. tobacco and vaping industry, proponents of vaping, influencers), and subsequently the platforms. For instance, the tobacco and vaping industry, along with proponents of vaping and influencers capitalised on high exposure, effective promotional opportunities, and the ability to engage and reach extensive audiences using relatively little effort and resources. In turn these actions had the potential to result in substantial revenue for social media companies. These industries were identified as the “*power in the marketplace.”* One participant stated.

*… the only entities that really have a global lens on social media trends and regulation are the* [social media] *companies themselves.… See, the earth is basically one country in their mind.”* (Participant 13: Global public health policy; UK university).

Several participants highlighted the consequential opposition that social media companies and other interested actors would enact, both directly and indirectly to avoid outside regulation. For example, “[opposition] *from the people who are doing things on Instagram… But there’ll be much stronger opposition from the companies behind them, the industries behind them and other related industries.”* (Participant 4: Public health; Aus university).

##### Self-regulation

Current self-regulation of platform content by social media companies was broadly reported as hindering the ability to adequately manage e-cigarette content. Self-regulatory models were predominantly viewed as inappropriate, problematic, not workable and “*in favour of industry”* [social media companies]. Overwhelmingly, participants highlighted the ineffectiveness of self-regulation in this space, *“Self-regulation is just ultimately, you know, largely a failure… There’s no incentive to do it.”* (Participant 11: Public health; US university).

Participants suggested that platform self-regulation was inadequate in ensuring e-cigarette content moderation occurred, as platforms could choose when to enforce, or not to enforce, even their own content policies:

*They don’t even get slaps on the wrist typically. And as a result, we’re seeing not enough done about this kind of content that is very harmful.”* (Participant 2: Law and social media; Aus university).

The reported lack of repercussions for this behaviour contributed to perceptions that the companies were ungovernable. Participants suggested that social media companies perpetuated this notion to further a self-regulation agenda to policymakers, who were viewed as lacking the knowledge needed to make reforms and were slow to regulate:

*“This idea that we would allow a newspaper or a billboard company to self-police and then just go oh, that’s a shame when the self-policing didn’t work, it’s nonsense. So why do we allow that to happen with digital media companies? Because our lawmakers are old and tired and can’t figure out how to regulate these platforms? It’s just lazy.”* (Participant 5: Public health; Aus university).

However, there was minority support for some degree of self-regulation using a stepwise approach:

*So firstly*, [social media companies] *write a* [self-regulatory] *code*. *If your code is strong enough, then we’ll let you run with it.…And if you fail, we’ll make it mandatory, and your failure will lead to pecuniary penalties.”* (Participant 8: Governance and telecommunications; Aus university).

##### Opacity

Participants commented on the challenges of distinguishing between organic, or paid/commercial content, reporting that different types of content can influence opinions as to how this content should be managed. Participants suggested that individuals produced organic content to present opinions, while commercial content was supported by vested interests and represented potential financial gain. Organically produced content by individuals was seen as “*a much greyer area”*.

*Popular forms of expression about or representation of vaping by ordinary social media users, by musicians, artists, celebrities and so on. I mean, you can’t ban that in the sense that you can’t ban people from posting images of themselves smoking or drinking or doing other stupid things*.” (Participant 9: Digital media; Aus university).

The commercial benefits that influencers gain from sharing content on their accounts means they need to use appropriate hashtag terms (e.g., #ad, #sponsored and #gifted) to identify partnerships and renumerations in their posts. However, participants questioned the effectiveness of terms such as ad and sponsored in drawing adequate attention to the incentives and payments received, as these hashtags were not noticeable in the context of the post:

*I personally am not convinced that #ad is sufficient to bring full attention to* [social media] *users that it might be a paid promotion.”* (Participant 12: Consumer advocacy; Aus NFP).

Participants also questioned whether the use of hashtags was ultimately just a way around policy and regulation.

*It’s a form of commercial communication. It’s an advertisement.”* (Participant 5: Public health; Aus university).

There was also commentary by participants regarding known influencer activity, with one participant stating:

*I know for a fact that influencers are being used to promote these products* [e-cigarettes] *100%. Like you’d have to be very naive to not think that’s happening.”* (Participant 5: Public health; Aus university).

#### Addressing complexity

**Fig. 2:**
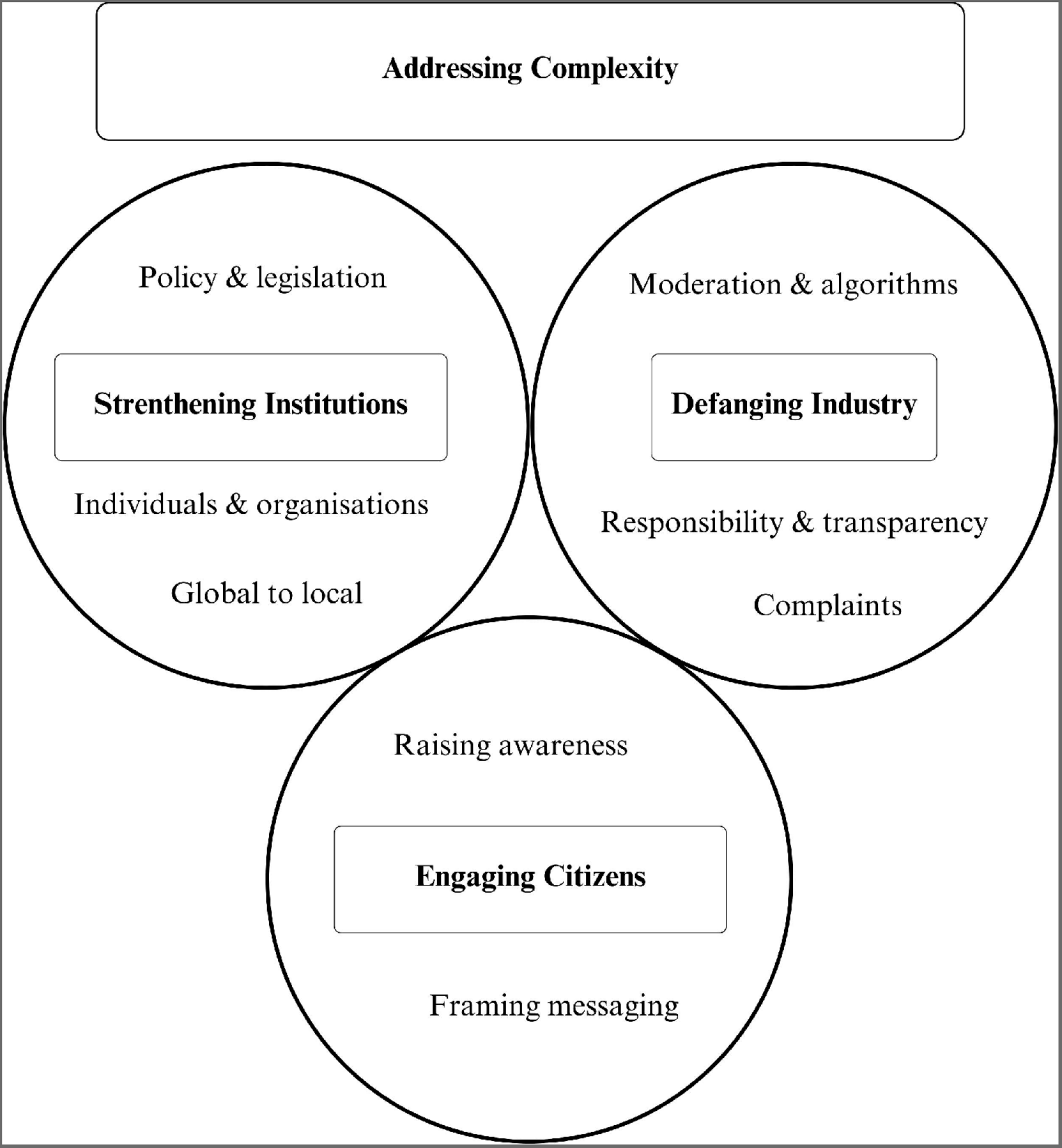
Addressing complexity (theme and subordinate themes)

Participants described potential strategies to address the perceived complexities of the social media landscape as it related to e-cigarettes. The subordinate themes were Strengthening Institutions (global to local, policy and legislation, individuals and organisations); Defanging Industry (responsibility and transparency; moderation and algorithms; complaints); and Engaging Citizens (raising awareness, framing messaging), as presented in Fig. 2.

#### Strengthening institutions

##### Global to local

The management of social media content was seen as a global issue, requiring a global response. Narratives highlighted the importance of cross-border collaboration and international cooperation through government involvement and leadership, to enable appropriate management of e-cigarette related content on social media.

The WHO was nominated *“as an engine and driver”* to lead global action through its FCTC, with reference to Article 13 (which lays down basic obligations to Parties to ban tobacco advertising, promotion and sponsorship).

*But it’s only going to really be effective* [management of e-cigarette related content on social media] *and happen, if government bodies and global cooperation through the* [WHO] *FCTC actually make it illegal and charges these media companies for violating the law.”* (Participant 5: Public health; Aus university).

Conversely, one of the participants had a conflicting opinion of the WHO FCTC, believing its position had been weakened, stating that “*it* [WHO] *has been relatively defanged.”* (Participant 11: Public health; US university).

Some participants argued that although social media companies are global businesses, they operate in regions and “*have the capacity to adapt their offerings within jurisdictions based on regulatory standards and posture of regulators.”* (Participant 12: Consumer advocacy; Aus NFP). Cases were cited where social media companies had operated to remove certain content based on the requirements of specific regions. For example, the eradication of alcohol sales on eBay (sales of alcoholic beverages is only allowed by pre-approved sellers who hold a valid liquor licence and buyers older than 18 years of age). Other cited examples of local action at a country-level, included the following.

*I think it’s totally possible for nations to have national-level frameworks that control what platforms can and can’t do. And again, I think the EU* [European Union] *with the General Data Protection Regulation* (GDPR) *has really shown us you can do that, and Australia has as well around things like the News Media Bargaining Code.… you can set national rules for platforms if you’re a valuable market and they will comply with them.”* (Participant 9: Digital media; Aus university).

##### Policy and legislation

Participants called for appropriate legislative change to enable the management of social media companies and in turn the management of their content. Existing content policies driven by social media companies could reportedly be “*easily wormed out of*,” while legislation was suggested as far more powerful.

*Well, we need a regulatory framework to cover the social media platforms. I don’t think policies… they might have of their own, that you know, prohibits sale and prohibition of tobacco products and e-cigarettes, is sufficient, because it’s not being complied with. And so, you need actual laws and rules and enforcement mechanisms to make sure that they follow suit.*” (Participant 12: Consumer advocacy; Aus NFP).

However, participants emphasised that whatever legislation is introduced, the responsible governing body or organisation must have the authority, and resources to monitor and enforce legislation and “*the power to penalise”* to make social media companies comply.

*… the only way you’re gonna stop these companies is with massive financial fines and public naming and shaming, so you need to name and shame - who violated it, the processes, how much they were fined. And they need to pay those fines. And look, I have no concept of how much money is a lot of money. Is a million dollars a lot of money?… Is a billion dollars a lot of money?”* (Participant 5: Public health; Aus university).

##### Individuals and organisations

Participants recognised a range of individual and organisational actors that could and should play a role in managing social media content, whether that be through advocacy, oversight or legislative controls. Participants strongly emphasised the central role of government in stewarding this issue.

*… it’s a government responsibility to me. You know that, just like it is for all of our other tobacco control laws.”* (Participant 10: Tobacco control: UK NFP).

Participants spoke of the need for government leadership and political actors in the areas of communications, health and business. In addition, there were several government and government statutory organisations that were nominated as needing to play a current or future role in social media content management, in Australia, as cited below:

*We’ve got a group of regulators*, [such as] *the eSafety Commissioner, the Australian Communications and Media Authority* [ACMA], *the Office of the Australian Information Commissioner, which is the Privacy Commissioner and the ACCC* [Australian Competition and Consumer Commission] *have a working group* [working group on digital platforms], *which is basically looking at platform issues.”* (Participant 8: Governance and telecommunications; Aus university).

Other organisations nominated to oversee social media content, included the Australian Therapeutic Goods Administration (TGA) and local and federal departments of health. Participants argued that any organisation charged with this responsibility required appropriate levels of power to be effective. Participants also acknowledged the importance of intersectoral collaboration, which would bring together various actors with assorted knowledge and skills to inform discussions and action for better management of social media content, including:

*… people who are working in the social media, communications, and public health space would be a good point of departure, and then lawyers who are active in communications laws, laws pertinent to communications.”* (Participant 3: Public health and social media; US university).

#### Defanging industry

##### Responsibility and transparency

Participants noted that social media platforms have the capacity to remove accounts when they violate their content policies. Systematic enforcement of this approach was recommended with one participant suggesting, “*anyone who breaches it* [content policy]… *kick them out of social media permanently…”* (Participant 2: Law and social media; Aus university).

It was suggested that “*buy-in”* from companies regarding any strategy to better manage their social media content would be beneficial as “*they are extremely powerful, politically and economically.”* (Participant 12: Global public health policy; UK university). There was also recognition by participants that social media companies were facing lawsuits in the US due to their failure to manage content. These lawsuits may act as an incentive or catalyst for them to be more proactive in managing the content on their platforms. Some participants recommended consulting with social media companies to determine *how* they were going to manage content, and consultations with jurisdictions that had introduced controls:

*You know the EU is probably the strongest example of that, whereas they might not have done something particularly around vaping, they’ve done lots around putting requirements on platforms to monitor and manage certain kinds of content.”* (Participant 9: Digital media; Aus university).

Participants reported that adequate control of e-cigarette related content on social media would require transparent action by social media platforms for all types of content, including advertising, which participants suggested can be ephemeral. For example, participants cited the example of Facebook where it had tried to be more transparent with political advertising:

*They only do it for political adverts, but I think having some kind of function where you could actually go and search for sponsored content generally would seem like a potential goal to reach. So at least you can see the amounts they reach by category.”* (Participant 13: Global public health policy: UK university).

##### Moderation and algorithms

Participants suggested that social media platforms should continue using and expanding the role of people moderators to manage content. Currently, moderating processes are used by social media companies, but individuals expressed the need for this activity to be more focused and better resourced. For example.

*… content moderators are real people sitting in suburban Manila who are watching video material and photo material as it goes up, so you could do that.* (Participant 8: Governance and telecommunications; Aus university)

Social media companies also develop algorithms (a set of rules to be followed in calculations or problem-solving for computers) to manage their content. Participants suggested algorithmic moderation approaches as a practical approach to the management of social media content. Narratives also suggested a perception that algorithms could provide a mechanism to ensure the identification and removal of content or prevent certain content from ever existing. There was the suggestion that “*some of the best”* software engineers work at these social media companies, who hold suitable expertise to effectively manage content:

*Detecting vaping using algorithmic approaches is likely to be more successful than some other things that you want to get rid of… just detect it and delete it, it’s not an unreasonable first bit to do.”* (Participant 8: Governance and telecommunications; Aus university).

##### Complaints

There were recognised opportunities for maintaining and extending complaint systems at the individual and organisational levels. For example, participants noted that at the individual level community members could challenge or complain directly to the social media account holder who posted the content.

*There was an influencer who didn’t display #AD. I wrote on his Instagram. Hey, you didn’t put #AD or #SP* [sponsored], *with a smiley face. So, he deleted my comment and changed his thing within like an hour.”* (Participant 2: Law and social media; Aus university).

Alternatively, participants suggested that complaints could be made to the social media platform by “having a simple social media user complaint system that says I’ve seen this content.” (Participant 8: Regulation and governance; Aus university).Participants also described the role of an independent complaint panel as used in other areas of public health, which was seen as having added benefit of addressing the limitations of self-regulatory, industry-led systems:

*The other thing that I think has been done in Australia in the context of alcohol, is the civil society have set up independent panels where people and users can send complaints to put pressure on the Government. Because industry-led or affiliated panels tend to be quite weak and will actually turn a lot of complaints away or dismiss them.”* (Participant 13: Global public health policy: UK university).

#### Engaging citizens

##### Raising awareness

Narratives suggested that to effectively raise awareness of e-cigarettes and their impact, public education, particularly with specific target groups, such as the general community and decision makers was required. Participants suggested that recommendations for better management of social media content could act as a catalyst for change without the community seeing such changes as *“a threat to their freedom”*, or politicians being concerned that it may “*cost them votes”.* For this to occur, participants noted the need for a strong understanding of social media content management, and the benefits of better content management including reduced community exposure to harmful products. This included the need for critical health and media literacy.

*So, for me, from a* [country redacted] *perspective, you need both regulation and awareness raising and critical analysis. So, I think we have to engage with the target audience. We have to engage with young people, and we have to make them aware of how these multinational corporations are trying to addict them and why these products are not, in reality, not the way that the image is being presented.”* (Participant 10: Tobacco control; UK NFP).

##### Framing messages

Communicating the need for improved management of harmful content on social media was also suggested by participants. This included using strategies employed by public health actors in tobacco and alcohol control, such as putting pressure on social media companies regarding inappropriate content and drawing attention to the vested interests of companies selling harmful products. In addition, participants suggested building on existing work by taking a combined approach to harmful products (including gambling, smoking, and ultra processed foods) and calling for an enforcement agency that addresses harmful products more broadly.

*“I think it’s very hard to look at vaping in isolation from other harmful product industries and the commercial determinants of health more generally. I think partly it allows you to build much bigger advocacy coalitions and tie into much bigger conflicts of interest.”* (Participant 13: Global public health policy: UK university).

These strategies were highlighted as providing an opportunity to gain the attention of a range of political actors and build advocacy opportunities. In addition, participants suggested positioning harmful products more strongly through a social justice lens, “*particularly regarding the rights of children and the right to health”* (Participant 13: Global public health policy: UK university) and as a contributor to noncommunicable disease through the promotion of harmful products:

*So, we need to be looking at e-cigarettes, tobacco, alcohol, unhealthy food and drink. You know, Coca-Cola, et al, who are really running rampant because they have the resources and the time, they can buy in the expertise to do so.”* (Participant 10: Tobacco control; UK NFP).

## Discussion

Social media has changed the way people communicate, interact and access information, goods and services, providing a forum for exposure to a range of imagery and opportunities that often do not occur in the ‘real world’ [29]. There has been aggressive promotion of e-cigarettes via social media, specifically targeting adolescents and young adults [30, 31], supporting the normalization of e-cigarettes and increased use [16, 17]. How to deal with this new borderless digital media environment is a public health challenge.

By speaking with people who identified as working in public health, digital media, law, governance, tobacco control and advocacy, and using e-cigarette content on social media as a case study, we identified the complexity of the social media environment and potential opportunities for controlling the ready exposure to harmful content, via the themes of strengthening institutions, defanging industry and raising awareness.

In considering how to deal with social media content, we need to acknowledge the structural power of tobacco, vaping and media companies and highlight their global capacity to influence individual and community behaviour, as well as policy and public health outcomes [32]. Responding to this dynamic and financially lucrative environment presents a range of challenges, particularly considering the power of social media companies, the limited experience of regulators, and relative sluggishness of regulatory action to catch up to technology [33].

Social media platforms provide a powerful, inexpensive, and pervasive marketing stage for products, generating revenue primarily by collecting user data and capturing their attention, which is then monetised through advertising services [29, 34]. There are currently 4.9 billion social media users worldwide [2], spending an average of 144 min each day online [35]. These exposures and interactions translate into significant dollars for digital platforms, with TikTok generating $350 million in revenue in 2022, and Facebook, Instagram, Twitter, and Snapchat together generating $205 million [35]. The global e-cigarette market is expected to grow to USD 28.17 billion by 2023, with the online distribution channels expected to register the fastest growth [36].

Considering the global operations of social media companies, our study participants recognized that coordination across borders is critical to the management of the platforms and their content [29]. Some participants highlighted the relevance and leadership opportunity of the WHO FCTC, specifically Article 13 [18], while others were less sure about this approach. FCTC Parties have recognized the challenges around monitoring and enforcing cross-border advertising, and have called for processes that more effectively facilitate global cooperation to ban cross-border advertising and sponsorship [37]. However, others in our study, particularly those from the social media area, suggested that content could be controlled at a country level.

Participants in our study provided examples of regulations that have already been implemented to manage social media companies and their content at a country level. These examples included the EU’s General Data Protection Regulation, considered one of the strictest privacy and security laws globally [38]; and the Australian News Media and Digital Mandatory Bargaining Code, which enables Australian news businesses to bargain with digital platforms regarding payment for news. Other examples include the UK Online Safety Act [39], which ensures social media platforms are held responsible for the content they host [40] and Australia’s recently introduced Public Health (Tobacco and Other Products) Act 2023 [9] which will aim to address the proliferation of e-cigarette advertising and promotional activities on social media. How this particular Australian legislation, proposed to commence in April 2024, is enacted, and its effectiveness remain to be seen. It will be crucial that this legislation is regularly reviewed to limit the development of loopholes and ensure it maintains effectiveness in the dynamic online environment [37].

These examples of current and proposed regulations have been introduced at a country level by government to manage social media companies and the content published on their platforms, demonstrating that these companies are governable. However, any government lead regulation needs to be accompanied by overseeing organisations that have access to the resources to monitor, enforce and appropriately penalise these companies for non-compliance [41]. Identifying these agencies is another step in the process of governance and will require continuing government leadership to ensure society and individuals are protected from harm in this online environment [29].

More specifically, those participating in our study stated that self-regulation was largely a failure, as the growing digital marketplace provided the ideal environment for those with vested interests to promote and distribute e-cigarettes and other harmful products globally, via organic and commercial content [42]. However, no matter what legal regulations are imposed on platforms their internal commitment to self-regulation will impact compliance [43]. These companies still need to have clear policies and community guidelines that set the rules for conduct, as presently they are often vague and unclear [43, 44].

Currently social media company policies are enforced via a mix of moderation processes that include outsourced workers reviewing content, machine learning tools that detect and remove content, and internal policy teams that set standards and oversee the processes [45]. These moderation processes can be challenging due to their inability to interpret language, and context and community standards, making it difficult to distinguish between problematic and permissible posts [46]. Nonetheless, it is important to have ongoing monitoring and evaluation of moderation processes to assess the viability of any of these actions.

Drawing on our findings, we call for open community dialogue about social media companies’ operations to increase awareness of their processes and impact. This dialogue needs to include regulators so that informed debate can lead to appropriate cultural change around expectations of the company’s behaviour and in turn the content they host. Open dialogue will enable increased awareness about e-cigarettes and potentially other harmful products, such as gambling, alcohol, and ultra processed foods. Accompanying this, community online media literacy education and resources could be introduced to enable increased awareness and knowledge of what content is permissible and the implications of interacting in the online environment [29]. Equipping people with the skills to critically evaluate online information, will reposition the media user as an active participant [47]. Providing an independent easily accessible community complaints systems will further enhance the role of individuals and the community in managing social media content.

### Limitations

Our study’s sample size of 13 may be considered a study limitation, however, it does provide a range of insights into the challenges and opportunities for management of social media content. In addition, social media is a dynamic environment and therefore recommended responses to better manage its content may change over time.

## Conclusion

Through qualitative insights, participants working in the areas of public health, digital media, law, governance, tobacco control and advocacy, identified a range of levers that could be enacted to decrease exposure to e-cigarettes and theoretically to other harmful content on social media.

The management of social media content was seen as a global issue, requiring a global response, with the narrative highlighting the importance of cross-border cooperation. However, at a country level, government oversight and actions are the priority. This should comprise the development of national-level regulatory frameworks, which have government leadership and appropriate legislation; and in the Australian context, identification of organisation/s with suitable levels of regulatory power and resources to monitor, enforce and penalise noncompliant social media companies. This activity should be further facilitated by an effective independent complaints panel and also an internal commitment from social media companies to protect their users from exposure to harmful content.

In parallel, participants also identified the need to raise community awareness regarding social media platform operations. In particular strategies are needed to increase digital literacy regarding harmful social media content in conjunction with framing messages to increase pressure on social media companies to improve the management of unwanted and harmful content. Social media companies need to take responsibility for content published on their platforms, as these platforms need to be safe environments that do not expose users to harmful products that can increase adverse health outcomes.

## Data Availability

No datasets were generated or analysed during the current study. The dataset is available on reasonable request to the corresponding author.

## Abbreviations

Aus: Australia
ACCC: Australian Competition and Consumer Commission
ACMA: Australian Communications and Media Authority
FCTC: Framework Convention on Tobacco Control
NFP: Not-for -profit
TGA: Therapeutic Goods Administration
UK: United Kingdom
US: United States
WHO: World Health Organization
